# Engineered cartilage utilizing fetal cartilage-derived progenitor cells for cartilage repair

**DOI:** 10.1038/s41598-020-62580-0

**Published:** 2020-03-31

**Authors:** Do Young Park, Byoung-Hyun Min, So Ra Park, Hyun Ju Oh, Minh-Dung Truong, Mijin Kim, Ja-Young Choi, In-Su Park, Byung Hyune Choi

**Affiliations:** 1 Department of Orthopedic Surgery, Ajou University School of Medicine, Suwon, Korea; 2 Cell Therapy Center, Ajou University Hospital, Suwon, Korea; 3 Department of Molecular Science and Technology, Ajou University, Suwon, Korea; 4 Department of Physiology, College of Medicine, Inha University, Incheon, Korea; 5 Department of Radiology, Seoul National University Hospital, Seoul, Korea; 6 Department of Biomedical Sciences, Inha University College of Medicine, Incheon, Korea

**Keywords:** Translational research, Osteoarthritis

## Abstract

The aim of this study was to develop a fetal cartilage-derived progenitor cell (FCPC) based cartilage gel through self-assembly for cartilage repair surgery, with clinically useful properties including adhesiveness, plasticity, and continued chondrogenic remodeling after transplantation. Characterization of the gels according to *in vitro* self-assembly period resulted in increased chondrogenic features over time. Adhesion strength of the cartilage gels were significantly higher compared to alginate gel, with the 2-wk group showing a near 20-fold higher strength (1.8 ± 0.15 kPa vs. 0.09 ± 0.01 kPa, *p *< 0.001). The *in vivo* remodeling process analysis of the 2 wk cultured gels showed increased cartilage repair characteristics and stiffness over time, with higher integration-failure stress compared to osteochondral autograft controls at 4 weeks (*p* < 0.01). In the nonhuman primate investigation, cartilage repair scores were significantly better in the gel group compared to defects alone after 24 weeks (*p* < 0.001). Cell distribution analysis at 24 weeks showed that human cells remained within the transplanted defects only. A self-assembled, FCPC-based cartilage gel showed chondrogenic repair potential as well as adhesive properties, beneficial for cartilage repair.

## Introduction

Tissue-engineered cartilage (TEC) constructs aim to improve current cell therapy for articular cartilage defects such as autologous chondrocyte implantation by direct transplantation of engineered cartilage tissue. Expected benefits include direct cartilage formation by prefabricated hyaline cartilage-like tissue containing viable cells, theoretically providing better resistance towards the knee’s highly loaded environment^1,2^. Despite its sound concept, few TEC products have reached clinical trials^1^. Significant hurdles towards translation include limit in size, poor integration, and suboptimal microstructural biomimicry of native cartilage^1,2^. Scaffolds which are commonly used in TECs often make the construct relatively rigid and fixed in geometry. Such constructs often necessitate additional fixation and substantial debridement of the defect, often resulting in gap formation, damage to normal host tissue, and overall, increasing the difficulty of the surgical procedure^3^.

Scaffold-free engineering is an alternative approach that improves some previous limitations of TECs. Its relative plasticity enables them to be applied directly over the defects, minimizing gap formation and improving integration^4,5^. Despite reports of its feasibility and effectiveness, scaffold-free TECs must overcome certain limitations for successful clinical translation. First, a highly proliferative allogenic cell source is preferred, as scaffold-free engineering requires a relatively large number of cells, often difficult to obtain with autologous cells^6,7^. Secondly, scaffold-free TECs undergo considerable remodeling *in vivo*. Transplanted tissue should therefore predictably differentiate and remodel into hyaline cartilage with minimal dedifferentiation or cell loss^2^. Thirdly, TECs should adhere immediately to the defect site to obviate the need for additional fixation. Overall, current scaffold-free TECs utilizing chondrocytes and mesenchymal stem cells (MSC) have yet to provide a comprehensive answer to these limitations^2,4,5^.

In efforts to overcome these limitations, we first aimed to develop an engineered cartilage construct utilizing fetal cartilage progenitor cells (FCPC) via scaffold-free, self-assembly engineering. According to our previous work, FCPCs boast a high proliferative capacity through late passages and higher chondrogenic capacity compared to adult chondrocytes and bone marrow derived-mesenchymal stem cells (bmMSC)^8,9^. Furthermore, we have previously reported a scaffold-free, self-assembly method using passaged chondrocytes^10^. Secondly, we aimed to optimize the cartilage gel’s plastic and adhesive features that would lead to a fixation-free, seamless fit of the cartilage gel into the defect. Finally, we sought to analyze cartilage repair efficacy, remodeling process, and cell distribution of the cartilage gel after transplanation in a nude mouse model and a non-human primate cartilage defect model *in vivo*.

## Results

### Characterization of fetal cartilage progenitor cell-based gels relative to *in vitro* culture time

Characterization of FCPCs used in this study revealed surface marker expression similar to MSCs (Supplementary Fig. 1A, Supporting Information). FCPCs also showed doubling time ranging from 2~4 days until passage 15 and trilineage differentiation potential towards adipose, osteogenic, and chondrogenic tissue, similar to our previsouly published result (Supplementary Fig. 1,B,C, Supporting Information)^9^.

**Figure 1:**
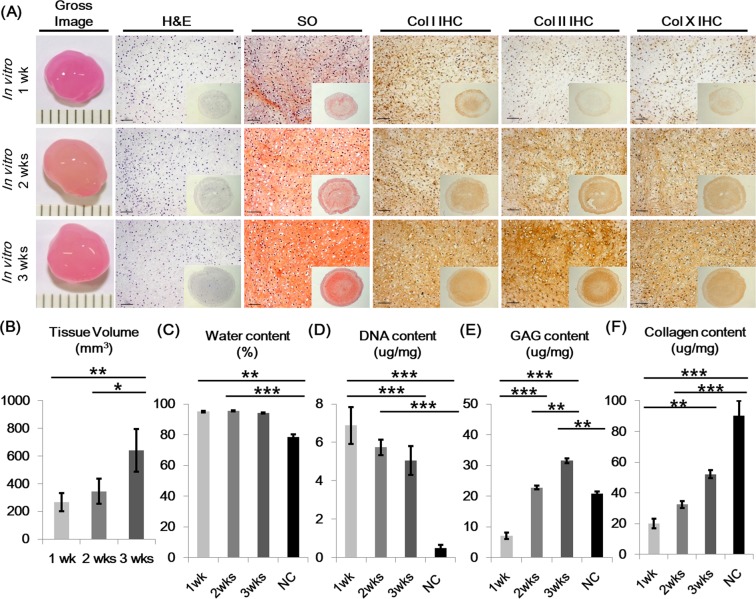
*In vitro* histologic and biochemical characterization of cartilage gels according to self-assembly time. (**A**) Gross image and histologic characteristics of cartilage gels. The overall construct size increased over culture time, as seen in gross images and tissue volume analysis (**B**). H&E staining results showed decrease in cellularity, while Safranin-O staining results showed increase in glycosaminoglycan content over culture time. Cartilage gel constructs also showed increased expression of chondrogenic differentiation markers such as Collagen type I, II, and X over culture time. Cartilage differentiation features were further analyzed with water (**C**), DNA (**D**), glycosaminoglycan (**E**), and collagen content (**F**) analysis, showing increase of cartilage like qualities over time. Statistical analysis done with Kruskal-Wallis test (*p < 0.05, **p < 0.01, ***p < 0.001). Magnification x30, x200. H&E; Hematoxylin and eosin, SO; Safranin-O, Col; Collagen, IHC; Immunohistochemistry, NC; Native cartilage.

**Figure 2:**
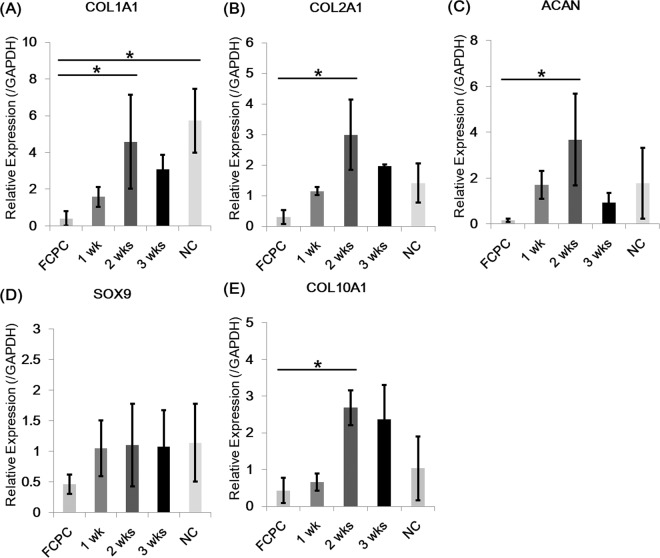
Real-time polymerase chain reaction analysis of chondrogenic markers in cartilage gels. The 2-week cultured cartilage gels showed significantly higher levels of extracellular matrix gene expression, COL1A1 and COL2A1, compared to fetal cartilage progenitor cell group (**A,B**). The 2-week culture group also showed increased expression of other chondrogenic markers, ACAN and SOX9, albeit without statistical significance (**C,D**). COL10A1, a chondrogenic differentiation marker, showed increased expression over culture time (**E**). Statistical analysis done with Kruskal-Wallis test (*p < 0.05, **p < 0.01, ***p < 0.001) and number of biological replicates n = 5. FCPC; fetal cartilage progenitor cell, NC; Native cartilage.

Cartilage gels were engineered with FCPCs using a previously described, scaffold-free self-assembly method, which include a high-density culture phase, followed by a self-assembly-differentiation phase^10^. Self-assembly, differentiation culture was carried out in three different durations of 1, 2, and 3 weeks, using defined media without exogenous growth factors. The effects of differentiation culture duration were analyzed. Cartilage gels overall showed increased chondrogenic features relative to *in vitro* differentiation culture time. Tissue volume, GAG content, and collagen content increased over time (Fig. 1A,B,E,F). Cellularity decreased over time, while water content of cartilage gels remained significantly higher than native cartilage until 2 weeks (Fig. 1A,C,D). By week 2 (2-WCG), GAG content showed no difference to native cartilage (NC) (Fig. 1E). The gene expression profile for 2-WCG showed increased expressions of COL1A1, COL2A1, ACAN, and COL10A1 compared to 2D cells (Fig. 2).

### Biomechanical properties of fetal cartilage progenitor cell-based cartilage gels

**Figure 3:**
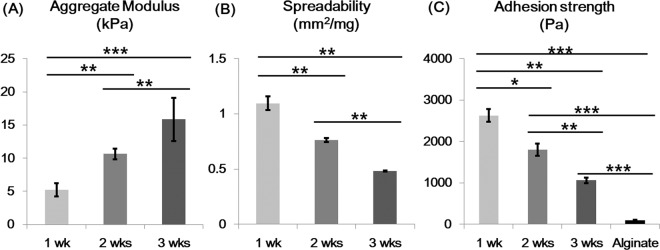
Biomechanical properties of cartilage gels. (**A**) The aggregate modulus increased over the period of *in vitro* culture time. (**B**) Spreadability, the measurement of spread area of cartilage gel under a fixed load, showed an inverse relationship to the aggregate modulus results, with the 1 week group being the most plastic. (**C**) Adhesive strength analysis also showed a decrease in adhesive strength over culture time. All cartilage gel groups showed significantly higher adhesive strength compared to alginate gels. Overall, cartilage gels showed both plastic and adhesive features, which may be useful during transplantation. Statistical analysis done with Kruskal-Wallis test (*p < 0.05, **p < 0.01, ***p < 0.001).

Cartilage gels were designed to be directly applied on irregularly shaped cartilage defects without the use of adhesives. Associated key biomechanical properties of stiffness, plasticity and adhesiveness were analyzed. Plasticity and adhesiveness showed an inverse relationship to stiffness, according to culture duration (Fig. 3). Adhesion strength was higher compared to alginate gels with the 2-WCG showing a near 20-fold higher strength (Fig. 3).

### Selection of cartilage gels for *in vivo* transplantation

The 2-WCG gels among all groups were selected for further analysis, considering the concurrent chondrogenic features and biomechanical properties of plasticity and adhesiveness (Supplemental Videos 1 and 2 of Supplemental Data).

### Remodeling process of the 2-week group cartilage gels after transplantation in human cartilage defects within a nude mouse model

**Figure 4:**
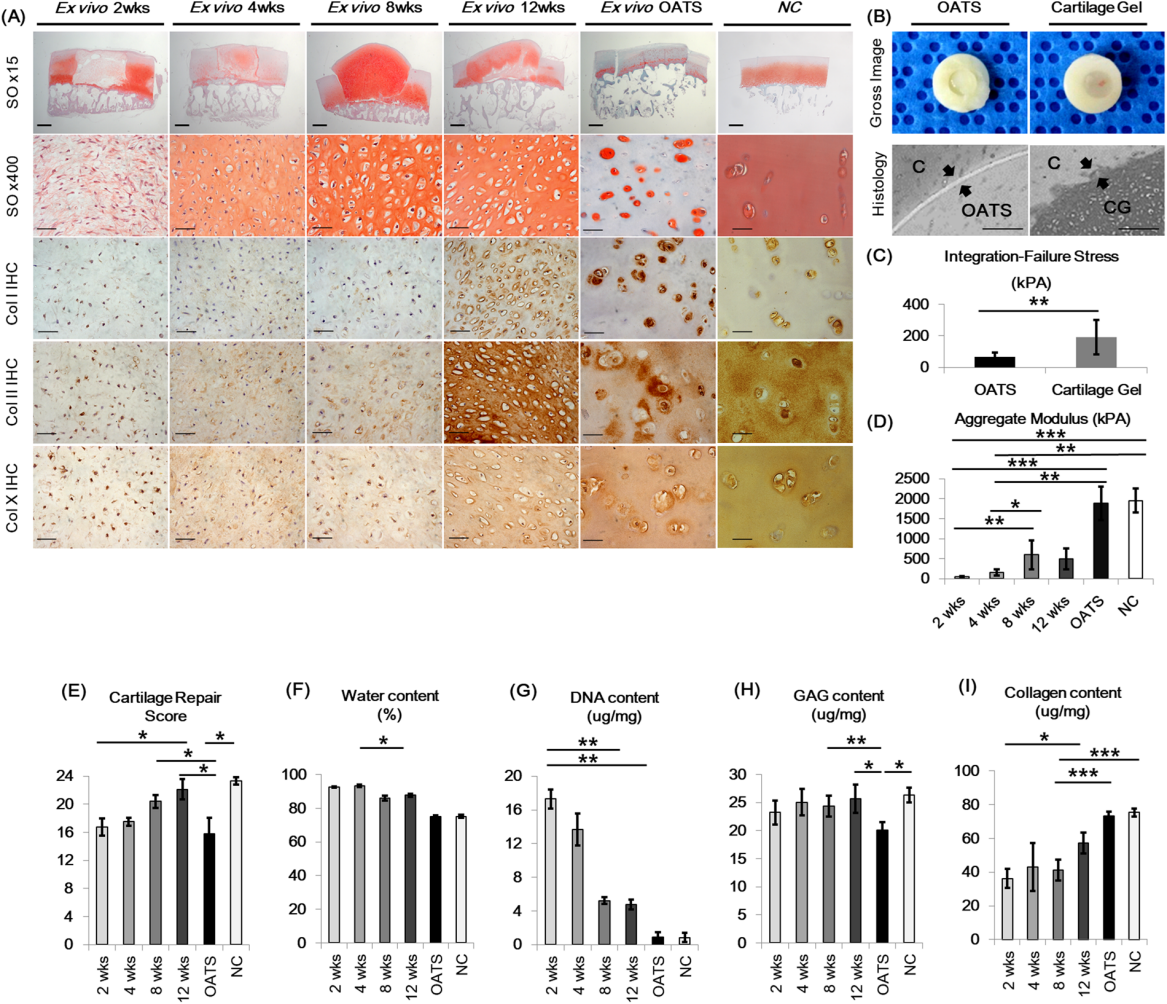
Transplantation of 2-week cultured cartilage gels in a nude mouse cartilage defect model. (**A**) Histological analysis of transplanted cartilage gel. Cartilage gels continued to differentiate after transplantation, as seen by the increase in safranin-O staining intensity, as well as increase in collagen type I, II, and X staining area. Cartilage lacunae was seen as early as 4 weeks. Cartilage gels integrated well with surrounding cartilage. (**B**) Cartilage-to-cartilage integration was further analyzed using cartilage cylinder constructs and cultured *in vivo* for 4 weeks. Cartilage gels integrated well into adjacent cartilage and showed significantly higher integration failure stress compared to OATS group. Black arrows indicate cartilage to implanted construct interface (**C**). (**D**) Transplanted gels showed increase in aggregate modulus through 8 weeks post-transplantation. (**E**) Cartilage repair scores after transplantation of gels improved over time. Decrease in water content (**F**), DNA content (**G**), and increase in collagen content (**I**) over time shows further cartilage-like differentiation of the gels after transplantation. (**H**) Glycosaminoglycan content of the gels remained higher than cartilage. Overall, transplantation of the 2-week cultured cartilage gels in an *in vivo* cartilage defect model showed time dependent cartilage differentiation and excellent integration with surrounding cartilage. Statistical analysis done Kruskal-Wallis test (**D**~**G**,**I**) and Mann-Whitney test for (**H**) (*p < 0.05, **p < 0.01, ***p < 0.001). Magnification x15, x400. OATS; Osteochondral autograft transplantation, GAG; glycosaminoglycan, C; Cartilage, CG; Cartilage gel, NC; Native cartilage.

The 2-WCG gels were transplanted into human full-thickness cartilage defect osteochondral blocks within a nude mouse (athymic NCr-nu/nu) model (n = 10 mice/group) to evaluate *in vivo* remodeling process of gels. Postoperative time points of 2, 4, 8, and 12 weeks (POD 2, 4, 8, 12 wks) were analyzed, with 8 week osteochondral autologous transplantation (OATS) blocks and NC serving as controls. Overall, cartilage gels showed continued differentiation after *in vivo* transplantation, expressing increased hyaline-like cartilage qualities over the 12 week time period, with 8-week samples showing lacuna formation (Fig. 4A). Cellularity and water content of transplanted cartilage gels decreased over time, while GAG content of the neo-cartilage remained higher than NC (Fig. 4A,F,G,H). Total collagen content increased over time, with IHC showing increases in collagen type I, II, and X expression (Fig. 4A,I). Stiffness of neo-cartilage increased over time until POD8 wks (Fig. 4D). Cartilage gels also showed significantly higher integration failure stress compared to OATS group (Fig. 4B,C). O’Driscoll cartilage repair scores, which evaluates components of cartilage-like tissue characteristics, structural integrity, and freedom from degeneration, also improved over time (Fig. 4E)^11^.

### Cartilage repair of the constructs transplanted on non-human primate cartilage defect models

**Figure 5:**
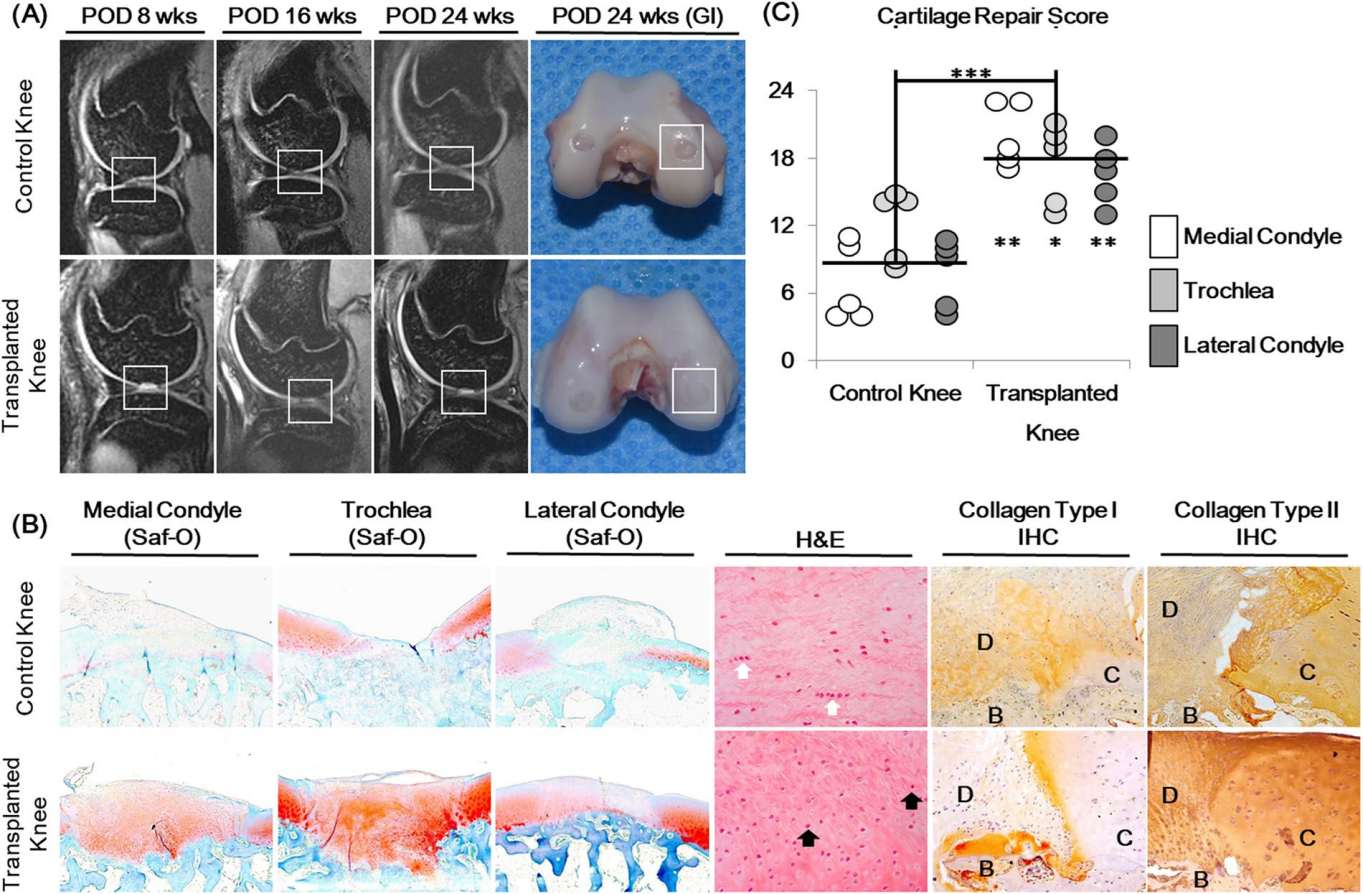
Cartilage repair analysis of 2-week cultured cartilage gels transplanted in a non-human primate cartilage defect model. (**A**) MRI images of femoral condyles taken at 8, 16 and 24 weeks after surgery along with final gross images taken at 24 weeks showed continued filling of the cartilage defect. (**B**) Histological analysis of cartilage defects 24 weeks after surgery showed better cartilage repair in cartilage gel groups, compared to defect only control groups. Repaired cartilage of the cartilage gel group showed lacunae formation (black arrow), and absence of cell clustering present in the control group (white arrow). Repaired cartilage expressed similar levels of collagen type I and II to surrounding hyaline cartilage. (**C**) Dot plot of cartilage repair score results. Cartilage repair scores were significantly better overall and in each anatomic location of the defect compared to the control group. Statistical analysis done with Mann-Whitney test (*p < 0.05, **p < 0.01, ***p < 0.001). Magnification x15, x100, x200. GI; Gross image, H&E; Hematoxylin and eosin, SO; Safranin-O, IHC; Immunohistochemistry, D; Defect, C; Native Cartilage, B; Bone.

Cartilage gels were transplanted in full-thickness cartilage defects of non-human primate knee joints (5 animals, 10 knee joints). Cartilage gels were transplanted on the left knee joint (transplanted knee), within three defects made in the trochlea, medial, and lateral condyle cartilages of the femur. Empty, full thickness defects made in the right knee joint (control knee) served as controls. MRIs were taken at POD 8, 16, and 24 wks to follow the healing process, and animals were euthanized at POD 24 wks for tissue harvest. All defects in the transplanted knees remained filled through the 24 wk follow-up period (Fig. 5A). At POD 8 wks, defects within the transplanted knees showed filling with high signal intensities whereas defects within the control knees showed irregular margin defects void of signal intensities in MRI (Fig. 5A). Histology of repaired cartilage showed intense safranin-O staining and integration with surrounding tissue in the transplanted knee defects (Fig. 5B). Cellularity was higher in the transplanted defects without cluster formations (Fig. 5B). IHC showed more intense collagen type II and weaker collagen type I staining in the repaired cartilage of the transplanted knees compared to control knees, similar to adjacent hyaline cartilage (Fig. 5B). O’Driscoll scores for the transplanted defects were better than control defects at all sites^11^.

### Cell distribution analysis of the cartilage gels after transplantation

**Figure 6:**
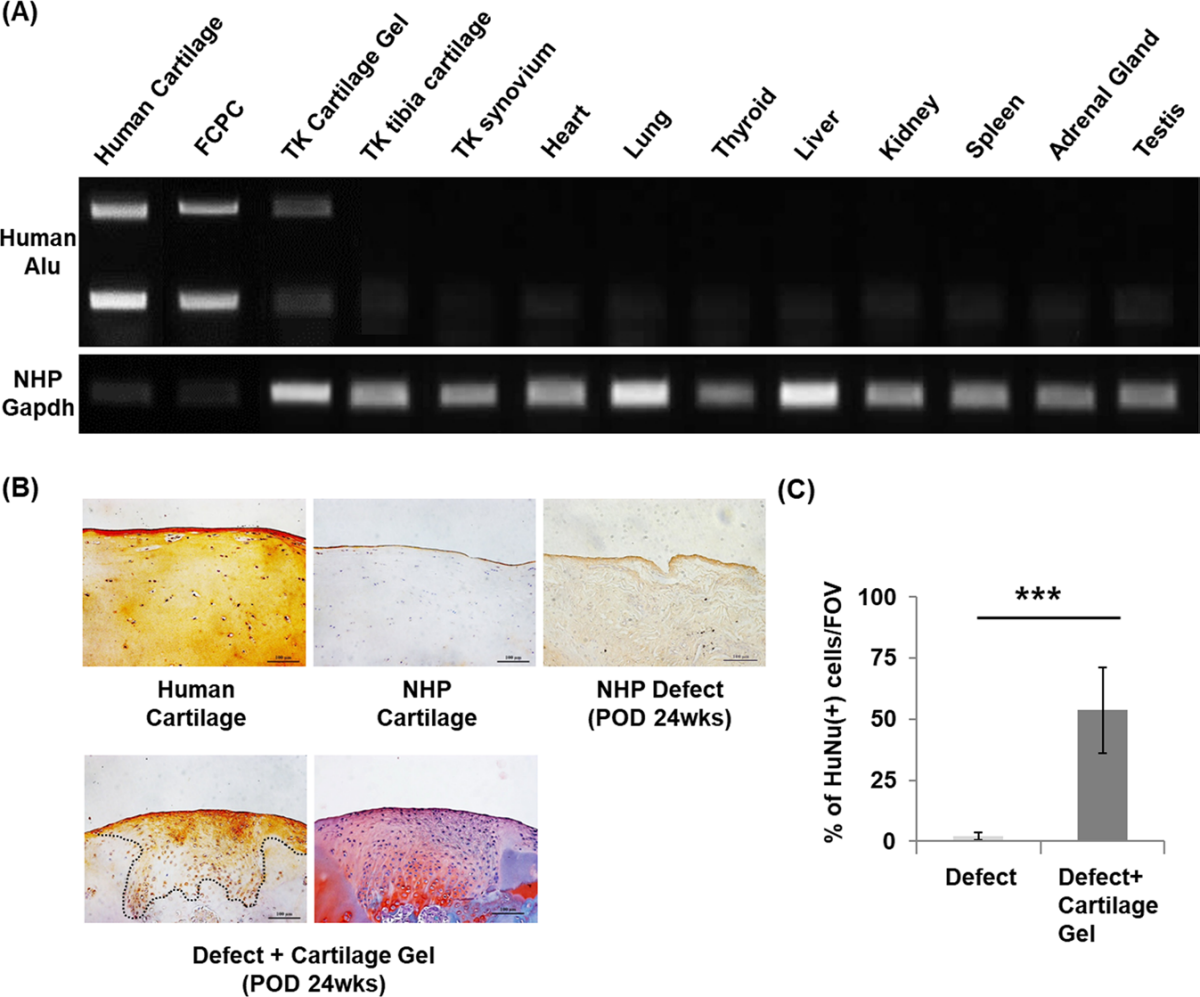
Cell distribution of transplanted cartilage gels in a non-human primate cartilage defect model after 24 weeks. (**A**) Representative RT-PCR result bio-distribution using human specific Alu sequence. Human Alu was detected in the transplanted cartilage tissue and was absent in adjacent tibial cartilage, synovium, and other vital organs. (**B**) Cell tracking using human anti-nuclear antibody. Immunohistochemical staining results showed that cartilage gels of human origin remained within the defect after 24 weeks. Safranin-O slide shown as reference for repair site. (**C**) Semi-quantification of immunohistochemistry results showed over 50% of human anti-nuclear antibody (+) cells within the defect. Statistical analysis done with Mann-Whitney test (*p < 0.05, **p < 0.01, ***p < 0.001). Magnification x40. FCPC; fetal cartilage progenitor cells, TK; transplanted knee, NHP; non-human primate, HuNu; human anti-nuclear antibody, FOV; field of view (x40).

Cell distribution of the gels after 24 weeks showed transplanted cells remaining within the defect. RT-PCR of human specific ALU sequences showed human ALU expression in the repaired cartilage of the transplanted knee (Fig. 6A). Human ALU was not expressed in other intra-articular structures of the transplanted knee including ipsilateral tibial cartilage and synovium, nor in other major organs. IHC analysis using human specific anti-nuclear antibody concurrently showed expression within the repaired cartilage in transplanted knees (Fig. 6B,C).

### Postoperative inflammation analysis

**Figure 7:**
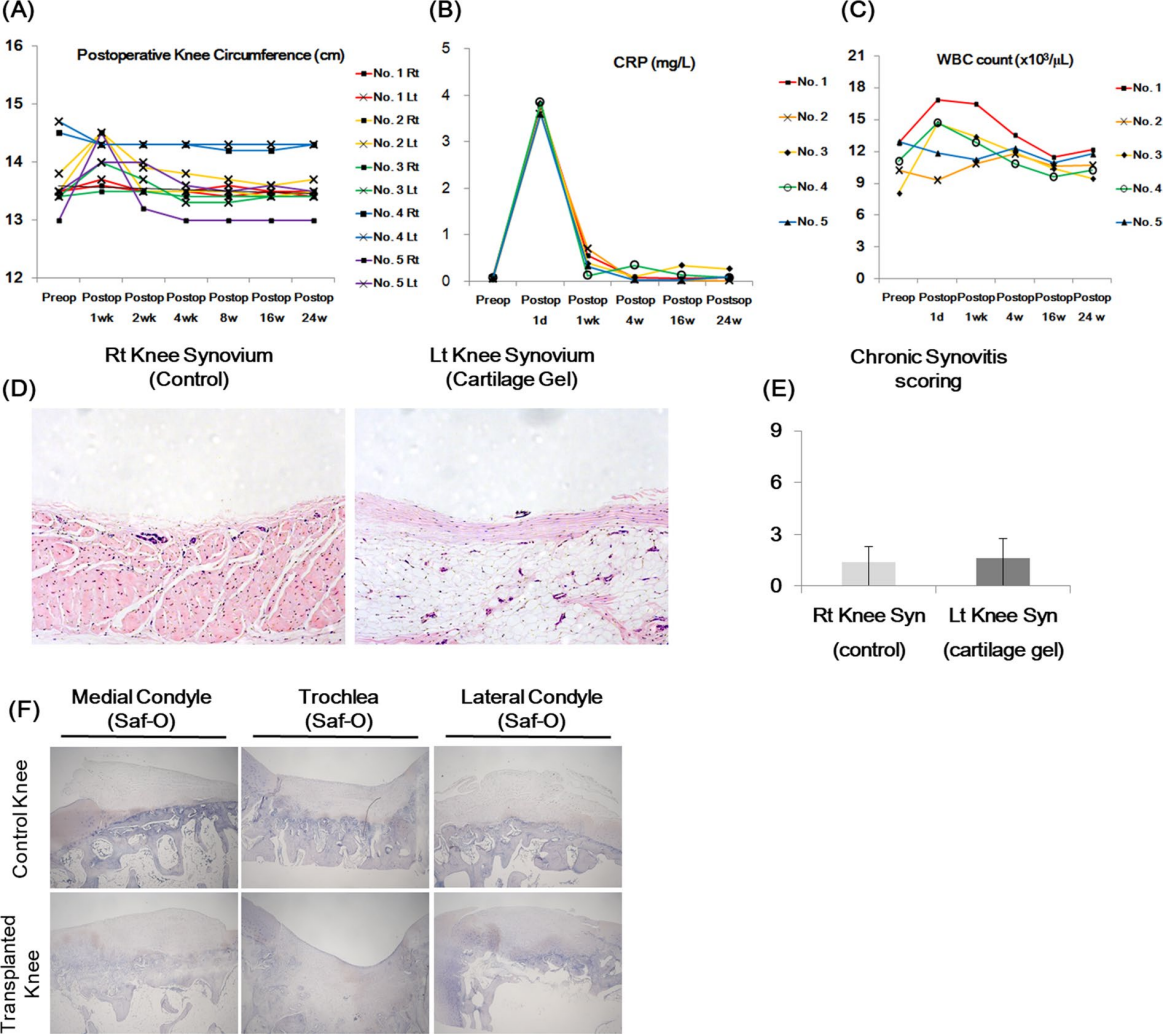
Postoperative inflammation analysis of non-human primate cartilage defect model after cartilage transplantation: Right (Rt) knees received defect surgeries as controls, while left (Lt) knees received cartilage gel transplantation. (**A**) Knee circumference was measured before and after transplantation in all five animals to assess indirect inflammation on the joints. Note that knee circumferences returned to baseline in all groups by postop week 4. (**B**) WBC count was measured from whole blood before and after transplantation to assess inflammation. Note that no leukocytosis/ leukopenia was observed in all groups beyond postop week 4. (**C**) C-reactive protein level was measured from whole blood before and after transplantation to assess inflammation. Note that all animals achieved levels below 1 mg/L by postop week 1. (**D**) Histology (H&E) of synovium. None to mild inflammatory reactions were observed in all synovium, with no difference between controls and cartilage gel transplantation groups **(E**). Overall, signs of rejection, or chronic inflammation were not present in all animals. (**F**) IHC stain for inflammatory marker CD45 was performed. Statistical analysis done with Mann-Whitney test (*p < 0.05, **p < 0.01, ***p < 0.001). Magnification x100, x40.

Postoperative inflammatory reaction was analyzed with gross examination, measurements of postoperative knee circumference, white blood cell (WBC) count, blood C-reactive protein (CRP) levels, synovial histology, and transplantation site IHC. On gross examination, no wound problems were noted in all animals on all observation time points. Postoperative knee circumference of the transplanted knees returned to baseline levels (Δ < 3 mm) in all animals by 4 weeks (Fig. 7A). WBC analysis showed no leukocytosis nor leukopenia beyond POD 4 weeks (Fig. 7B). CRP levels also increased over 3 mg/L in all animals at POD 1 day, yet decreased below 1 mg/L in all animals by POD 1 week (Fig. 7C). Synovial histology showed no significant differences between control and transplanted knees, with both showing not to mild inflammatory changes at POD 24 weeks (Fig. 7D,E)^12^. All defect sites were absent from CD45 expression as shown in the IHC figures of the defects (Fig. 7F). Overall, no signs of acute rejection or chronic inflammation were observed in all animals.

## Discussion

Several scaffold-free TECs have reached clinical trial stages with promising early results, with at least two products available on the market^1^. Chondrosphere® (co.don AG, Teltow, Germany) a cartilage tissue aggregate product using autologous chondrocytes, have shown some improvement in clinical scores^1,13^. Revaflex™ (Isto Technologies, St. Louis MO), a neocartilage product based on allogeneic juvenile chondrocytes, is relatively large (2.2–2.5 cm diameter) and stiff compared to other scaffold-free TECs requiring tailoring and fixation during implantation. A Phase I/II trial showed 67% of patients to have ‘normal to nearly normal’ cartilage repair one year postoperatively, without any adverse immune response potentially associated with allogeneic cells^14^. Despite early clinical success, we still have little scientific evidence regarding the optimal cell source, therapeutic mode of action, and optimal biomechanical properties for clinical translation. Our study challenged these issues by first utilizing a highly proliferative and chondrogenic cell source and secondly by optimizing clinically relevant biomechanical properties *in vitro*. Finally, the therapeutic mode of action was investigated through remodeling process and cell distribution analyses, showing that the construct remains within the transplanted defect in the long run, successfully remodeling into hyaline-like cartilage *in vivo*.

Scaffold-free TECs invariably require a relatively higher number of cells compared to scaffold based TECs^6,7^. With intention to treat by a single procedure, a highly proliferative allogeneic stem cells with chondrogenic capacity is desirable. FCPCs are easy to isolate and expand, resulting in high yields (6.5 ± 0.95 × 10^7^ cells/ 1 g tissue)^9^. Secondly, they possess stem cell characteristics of self-renewal, multilineage differentiation, and immune-modulation, with the potential to be a safe allogeneic cell source^9,15–18^. Thirdly, cartilage tissue formation ability of FCPCs are better than chondrocytes or MSCs, even without the use of exogenous growth factors. Previous studies of FCPCs have shown larger cartilage tissue formation with more GAG production compared to chondrocytes or bmMSCs^9,19,20^. Most importantly, tissue engineering with FCPCs may translate numerous regenerative benefits of fetal tissues. Fetal tissue responds to injury in a fundamentally different manner than adult tissue, resulting in a rapid, scar-less healing in many dense connective tissues^21,22^. This enhanced healing potential is intrinsic to the fetal tissue itself rather than the fetal environment, as the healing potential carries over into an adult host after transplantation^21^. Our findings support previously published FCPC characteristics, where FCPCs dependably differentiated towards cartilage without the use of growth factors and continued ECM production (Figs. 4A,G, 5B,C). ECM of the cartilage gels expressed similar levels of collagen type II compared to mature cartilage, both *in vitro* and *in vivo*, which is often difficult to achieve in adult MSC-based systems^23–25^ (Figs. 1A, 2B, 4A, 5B). Fetal cell-derived ECM is known to have positive effects on stem cell proliferation, differentiation, and biomechanical function compared to adult cell derived ECM^26^. Biomechanically, fetal cartilage ECM is unconstrained compared to adult ECM, allowing considerable cell migration and remodeling^11,23,27^. These ECM characteristics may have influenced the excellent construct-to-cartilage integration results, which is affected by cell migration and matrix accumulation at the interfacial zone^28^ (Fig. 4G,H).

The assumption underlying the efficacy of TECs is the continued physical presence of the transplanted construct within the defect together with ongoing remodeling towards cartilage tissue after transplantation. The fate of TECs after intra-articular transplantation, however, is largely unknown both in clinical and preclinical studies^1^. Previous cell tracking results after intra-articular cell transplantation show gradual decrease of transplanted cells over time^29,30^. A cartilage repair study utilizing human umbilical cord blood-derived MSC-hyaluronic acid gel composite in a rabbit model resulted in disappearance of human origin cells by the 16^th^ wk^31^. One of the strengths of this study is the demonstration of the TEC’s continued presence and differentiation after *in vivo* transplantation, as shown in our remodeling and cell distribution data, Time-dependent cartilage differentiation (Fig. 4) and presence of human origin cells filling the cartilage defect after 24 wks was observed in the non-human primate model investigation (Fig. 6). The persistence of the cartilage gels may have been due to several factors, including the high integrative potential of the cartilage gels (Fig. 4G,H), low-immunogenicity of FCPCs, genetic similarities of non-human primates to humans, and continued maturation of ECM^32^. Further studies are required to investigate the phenotype of transplanted cells and their functionality.

The self assembly and remodelling process of scaffold-free TECs is also an integral part of its mode of action. This process generally assumes that TECs undergo matrix secretion and development of collagen cross-links, yet this has seldom been demonstrated. During the *in vitro* engineering period, N-cadherin activity is known to play a significant role in the initial cellular aggregation and self-assembly period^33^. Previous *in vitro* studies utilizing chondrocytes and MSCs have observed GAG production increase after day 14, and collagen increase after day 28, while our constructs expressed both GAG and collagen at earlier time points (Fig. 1)^33,34^. Mechanism behind remodelling process after transplantation is also not well known. A previous report utilizing synovium derived MSCs showed partial chondrogenic differentiation of the scaffold-free TEC after transplantation even without predifferentiation. The mechanism of differentiation was not investigated in this previous study, postulated to be a result of local environmental factors^5^. Our nude mouse model results show continued construct differentiation *in vivo* over 12 weeks. Similar differentiation process may have been present in an intra-articular environment, as sequential MRI images of non-human primate knees show gradual signal decrease within defects closely resembling adjacent cartilage. The continued differentiation after transplantation may have been largely due to commitment of FCPCs towards chondrogenesis. Further studies are required to analyze the continued *in vivo* chondrogeneic differentiation mechanism of FCPCs.

Another goal was to optimize the TEC’s biomechanical characteristics for clinical translation. Ideally, a gel-type TEC construct would be clinically useful if it can immediately adhere to and seamlessly fill an irregularly shaped defect without additional fixation, while maintaining some stiffness to withstand the mechanical demands of the articular joint. Previous studies of TECs have suggested that longer *in vitro* culture periods result in more mature constructs, which in turn, may negatively affect integrative properties^35^. We have optimized the TEC’s clinically relevant biomechanical properties by *in vitro* culture duration and have found that TEC’s stiffness was inversely proportional to plasticity and adhesiveness relative to culture time (Fig. 3). Notably, the adhesiveness of the 2-WCG was 20-fold higher than alginate gels (Fig. 3C) and was enough to overcome gravity as well as considerable shaking in water (Supplemental Video 1 of Supplemental Data). Immediate adhesion of TECs into the defect is clinically useful, as it allows for an easier, possibly minimally-invasive procedure^36^. This process is different from integration to host tissue, as integration is partly mediated by cell migration and proliferation around the construct edge which invariably takes time. Factors affecting immediate adhesiveness of scaffold-less TECs are relatively unknown^4^. Previous studies have utilized various methods to enhance immediate adhesion, from fibrin glue to photopolymerization^37–40^. The cohesive strength associated with the degree of the cartilage gel’s viscoelasticity may have played a role in the stickiness of our constructs to the defect area^41^.

A number of limitations exist in this study. First of all, cell isolations were done before any clear anatomical distinctions of developing cartilage, thereby possibly leading to incorporation of mesenchymal cells from different tissue lineage such as bone. This may be the reason for Collagen type I and X expression observed in Fig. 1. Resulting cartilage tissue from FCPCs, however, was devoid of calcification and bone formation as shown in our previous study^9^. Secondly, biomechanical analysis of the repaired cartilage after nonhuman primate transplantation was not performed. This was due to the characteristics of nonhuman primates. Nonhuman primates are used when elucidation of immune compatibility is needed for human originating cell therapy or biomaterial products. Nonhuman primates, however, have relatively small knee joints comparable to mature rabbits, which make them susceptible to substantial error during biomechanical testing. Nevertheless, the compressive modulus of the cartilage gels analyzed in the nude mouse model showed increase over time even without physiologic joint loading conditions. Another important consideration is the availability of these cells and ethical issues concerning clinical use of fetal cells^9,42^. More than 10 clinical trials using fetal stem cell transplantation are currently being conducted world-wide^42^. Previous proliferation data of FCPCs and prior related studies suggest that fetal cells lead to a very large cell yields, diminishing the need for repeated harvesting. Further knowledge in FCPC chondrogenesis and advancements in cloning may lead to dedicated cell lines of fetal cells that may circumvent the need for harvest altogether.

## Conclusion

We have developed an adhesive and highly chondrogenic FCPC-based cartilage gel potentially useful for cartilage repair. Our study improves current cartilage tissue engineering strategies in using a highly proliferative and chondrogenic allogeneic cell source that actually remains and remodels within the defect in the long term. Our work also provides a framework for optimizing clinically relevant biomechanical properties of scaffold-less TECs through *in vitro* culture and provides scientific evidence regarding the mode of action of engineered cartilage, useful for future studies and related preclinical trials.

## Methods

### Cell isolation, culture, and characterization

All protocols involving human tissue were performed under the approval of Institutional Review Board of Ajou University School of Medicine (AJIRB-MED-SMP-10-268). Human fetal cartilage tissues were harvested from four abortus within 24 hours following elective termination (n = 4, M GA11 wks, M GA12 wks, M GA12 wks, F GA12 wks) after informed consent from respective guardians. Cells were isolated from the femoral head cartilage and expanded using a previously published protocol^9^. Cartilage tissues were minced into small pieces and treated with 0.1% collagenase type 2 (Worthington Biochemical Corp., Freehold, NJ, USA) in high-glucose DMEM containing 1% FBS at 37 °C under 5% CO_2_. After 12 h, the released cells were centrifuged at 1700 rpm for 10 min, washed twice, and cultured in DMEM supplemented with 10% FBS, 100 U/ml penicillin G, and 100 μg/ml streptomycin at a density of 8 × 10^3^ cells/cm^2^. When cells reached 80% confluence, the 0.5% Trypsin-EDTA of 10X to 1 × (Gibco, NY, USA) were used to detach the cells, which replated by the same way as above. Cells were expanded for two passages with culture medium changed every 3 days. All donor cells showed no significant difference in terms of morphology, proliferation, surface marker, senescence, and chondrogenesis as previously published^9^. Passage 2 cells were used for this study from all groups during *in vitro* experiments, and M GA11 wks cells were used for *in vivo* investigations.

Cells at passage 2 were analyzed for expression of stem cell related surface markers by flow cytometry. Briefly, cells in the suspension were incubated with anti-CD29-PE (Catalog No.

555443), anti-CD34-FITC (Catalog No. 555821), anti-CD45-PE (Catalog No. 555483), anti-CD73-FITC (Catalog No. 561254), anti-CD90-FITC (Catalog No. 555595), anti-CD105-FITC (Catalog No. 561443), anti-SOX2-PE (Catalog No. 560291) (BD Biosciences, CA, USA) and anti-OCT3/4-APC (Catalog No. IC1759A) (R&D Systems, MN, USA) for 40 min at 4 °C. Stained cells were determined by BD FACSCanto II flow cytometer (BD Biosciences, CA, USA) and analyzed using Flowing software (http://flowingsoftware.btk.fi).

For osteogenic and adipogenic differentiation, cells at passage 2 were plated in 6-well plates at densities of 2 × 10^3^ cells/cm^2^ and 2 × 10^4^ cells/ cm^2^, respectively. After 24 h, the cells were incubated in the differentiation medium for each lineage. The osteogenic medium consisted of α-MEM supplemented with 10% FBS, 10 mM β-glycerophosphate, 100 nM dexamethasone, and 50 μg/ml ascorbate-2 phosphate (Sigma-Aldrich, MO, USA). The adipogenic medium consisted of α-MEM supplemented 10% FBS, 1 µM dexamethasone, 10 μg/ml insulin, 0.5 mM isobutyl-methylxanthine, and 0.1 mM indomethacin (Sigma-Aldrich, St. Louis, MO, USA). After 3 weeks of differentiation, cells were stained with Alizarin red S and Oil red O (Sigma-Aldrich, MO, USA) to observe the degree of mineralization and the lipid droplets, respectively. For chondrogenic differentiation, 3 × 10^5^ cells at passage 2 were centrifuged at 500 × *g* for 5 min, and the cell pellet was cultured in a chondrogenic medium. The chondrogenic medium consisted of DMEM supplemented with 100 nM dexamethasone, 50 μg/ml ascorbate-2 phosphate, ITS supplement, 40 μg/ml proline, 1.25 mg/ml bovine serum albumin, 100 μg/ml sodium pyruvate (Sigma-Aldrich, MO, USA). After 3 weeks of induction, the samples were fixed with 4% formaldehyde and embedded in paraffin wax. Sections with a thickness of 4 μm were prepared and stained with Safranin O (Sigma-Aldrich, MO, USA) to observe the sulfated glycosaminoglycans.

We also examined the proliferation ability and phenotype of the FCPCs according to passage time. The doubling time of FCPCs was determined from passage 1 to passage 15 (n = 4). Cells were subcultured at 80% confluence. The doubling time was calculted using the following formula: DT = (T1 − T0)log2/(logN1 − logN0), where T1 − T0 = the culture period in days, N0 = the plating cell number, and N1 = the harvesting cell number. Accumulated cells numbers were calculated with passages or days.

### Fabrication of cartilage gels

Cartilage gels were fabricated using a previously published protocol^10^. Briefly, FCPCs were cultured in a high-density monolayer (at 2 × 105 cells/cm^2^) with DMEM supplemented with 100 nM dexamethasone, 50 μg/ml ascorbate-2 phosphate, ITS supplement, 40 μg/ml proline, 1.25 mg/ml bovine serum albumin, 100 μg/ml sodium pyruvate. They were cultured until full confluency two dimensionally when cells spontaneously formed a thin membrane. Subsequently, the medium was removed and 1X Trypsin-EDTA was added and incubated for less than 5 min at 37 °C. When the membrane was peeled off from the plate, the enzyme was immediately removed and the membrane was carefully isolated using a wide-bore pipette and moved individually to a 50 ml tube filled with 10 ml of defined medium without exogenous growth factors (high glucose Dulbecco’s modified eagle’s medium with an insulin–transferrin–selenium mixture, 50 mg/ml of ascorbate 2-phosphate, 100 nM of dexamethasone, 40 mg/ml of proline, and 1.25 mg/ml of BSA). Each tube was centrifuged at 100 × g for 20 min to consolidate the membrane into a pellet-type construct. The constructs were incubated for 16 h at 37 °C and then transferred to a 6-well culture plate for extended culture for 1, 2, or 3 weeks in a 37 °C humidified atmosphere of 95% air and 5% CO2. The culture medium (5 ml) was changed every 3 days.

### *In vitro* characterization of cartilage gels

After macroscopic examination, volume of the cartilage gels was measured using micro-CT (SkyScan, Kontich, Belgium). Scanning protocols are outlined in the Supporting Information Text^43^.

For histological analysis, cartilage gels were fixed in 4% formalin for 3 days and processed for tissue sectioning. Tissue sections of 4 µm in thickness were made and stained with hematoxylin-eosin (H&E), Safranin-O, and immuno-histochemical (IHC) analysis for type I collagen (1/100; Abcam, Cambridge, UK; catalog No. ab34710), type II collagen (1/100; Abcam, Cambridge, UK; catalog No. ab34712), type X collagen (1/100; Abcam, Cambridge, UK; catalog No. ab49945) using the DAB method.

For water content analysis, tissue samples were weighed (wet weight), freeze dried, and weighed again (dry weight). For glycosaminoglycans (GAG) content and DNA content analysis, samples were freeze-dried and digested with papain-digestion solution (5 mM L-cysteine, 100 mM Na2HPO4, 5 mM EDTA, and 125 mg/ml papain type III: Sigma-Aldrich, MO, USA). For papain digestion, the sample in 1 ml papain-digestion solution was incubated at 60 °C for overnight. The total GAG content was spectrophotometrically measured by using the 1, 9-dimethylmethylene blue colorimetric assay. Chondroitin sulfate sodium salt from shark cartilage (Sigma-Aldrich, MO, USA; catalog No. C4384) was also used as a standard^44^. Briefly, the assay was prepared via two steps: (i) The colour reagent was prepared by dissolving 16 mg dimethylmethylene blue in 1 l water containing 3.04 g glycine, 2.37 g NaCl and 95 ml 0.1 M HCl, to give solution at pH 3.0 (Sigma-Aldrich, MO, USA). (ii) The papain-digested GAG sample prepared as above was taken, and measured by reference to a standard calibration curve. The result was analyzed by Magellan v.6.4. software and Tecan microplate reader (Tecan Group Ltd, Männedorf, Switzerland). For total collagen content analysis, samples were measured by chloramine-T hydroxyproline assay^45^. Souluble peptides and proteins in each standard and tissue sample were hydrolyzed to individual amino acids by adding 500 μl of 4 N sodium hydroxide (NaOH) and incubating at 121 °C and 15 psi above atmospheric pressure for 20 min (using an autoclave). Samples were allowed to cool to room temperature and then neutralized with 500 μl of 4 N HCl. Hydroxyproline amino acids were converted to pyrolle-2-carboxylate by oxidation via addition of 0.625 mL of 0.05 M chloramine-T in 74% v/v H_2_O, 26% v/v 2-propanol, 0.629 M NaOH, 0.140 M citric acid (monohydrate), 0.453 M sodium acetate (anhydrous), and 0.112 M acetic acid (glacial), followed by incubation at room temperature for 20 min. Finally, 0.625 mL of 15% w/v DMAB (1 M) in 2-propanol plus concentrated acid (a.k.a. Ehrlich’s solution) was added to each sample and vortexed immediately to facilitate mixing. Samples were incubated at 65 °C for 20 min and then rapidly cooled by immersion in room temperature water to stop chromophore development.

Genetic markers were analyzed with real-time polymerase chain reaction for 1, 2, and 3 wk cultured cartilage gels, with FCPCs and mature cartilage tissue used as controls. All adult human cartilage and osteochondral tissue were harvested from morphologically normal (ICRS grade 0) lateral condyle osteochondral tissue obtained during joint replacement surgery. Protocols and primer information are outlined in Supporting Information and Supplemental Table S1.

Biomechanical analysis of cartilage gels was done by measuring the aggregate modulus, spreadability, and adhesive strength. For aggregate modulus, tissues were subjected to an unconfined compression test using Universal Testing Machine (Model H5K-T; H.T.E, Sanford, England). Each sample was placed on a bottom plate of the machine, and compressed at a speed of 1 mm/min. The machine was stopped automatically after moving a programmed length between the top and bottom plate^46,47^. The spreadability of cartilage gel was determined by pressing the tissue construct between top and bottom plates covered by paper, then 500 g standardized weigth was put on the upper plate and left for about 5 minutes, as previously described^48,49^. Diameters of spread circles were measured in mm and were taken as comparative value for spreadability. Adhesive strength of cartilage gels after transplantation into the human cartilage defect osteochondral blocks was measured using a push-out test as in previously described^50–52^. Briefly, The adhesive strength was evaluated as the forces at ultimate failure per unit of the interfacial area and determined using 5 mm in diameter of cylindrical-shaped indenter in an Universal Testing Machine fitted with a 5N maximum load cell. Alginate gels (2%) were fabricated using previously published methods and used for controls during adhesion strength testing^46,47^. All *in vitro* characterization analyses were done 5 times each.

### Remodeling process analysis using a nude mouse model

Full thickness cartilage defects of 3 mm diameter were made on 5 mm diameter human osteochondral blocks obtained from joint replacement surgery. Lateral femoral condyles blocks without gross cartilage wear were used. NCs were used as normal controls, obtained from lateral femoral condyles of fresh frozen cadavers without knee joint pathology. The 2-WCG gels were transplanted on the defects using a syringe without additional fixation. OATS was performed on osteochondral blocks as controls. Briefly, 3 mm diameter osteochondral defects were made with a matching size biopsy punch. The osteochondral block was gently reinserted into the defect similar to the OATS procedure using a plastic impactor (Arthrex, Naples, FA, USA). Osteochondral blocks were then transplanted subcutaneously into the back of nude mice (Athymic NCr-nu/nu, Koatech, Korea, n = 7 for each group of analysis), after institutional approval of Institutional Animal Care and Use Committee of Ajou University (IACUC No. 2014-0072)^53^. Osteochondral blocks were sampled at 2, 4, 8, and 12 weeks after transplantation and analyzed as described above. Histological images were scored for cartilage repair using O’Driscoll cartilage repair scoring system by two separate pathologists not involved in this study^54^. For cartilage-to-cartilage integration analysis, a human cartilage cylinder construct model was used as previously described^55^. Briefly, cartilage gels were implanted in the cylinder constructs and OATS was used as controls. Cylinder constructs were then subcutaneously transplanted into the back of nude mice (n = 7 for each group). The cartilage-to-cartilage integration was evaluated by safranin-O stain and mechanical test for integration strength using using a push-out test as in previously described^55^. A custom 2 mm in diameter of cylindrical-shaped indenter affixed to a Universal Testing Machine (fitted with a 5N, 100N, 500N maximum load cell) pushed the cartilage repair out of the cartilage annulus (1 mm/min) while recording load. Failure stress (integration strength) was calculated as the quotient of the load at failure and the interface area.

### Nonhuman primate cartilage defect model investigation

All protocols involving nonhuman primates (NHP) use were approved by Institutional Animal Care and Use Committee of Seoul National University (SNU-13-0251). All experiments were performed in accordance with relevant guidelines and regulations. A total of five skeletally mature male NHPs (*Macaca fascicularis*) aged 54 to 69 months weighting 4–5.5 kg were used for this study. Procedures for anesthesia and surgery for femoral chondral defects on both knees followed a previously published protocol and outlined in the Supporting Information Text^56^. Postoperative knee circumference was measured preoperatively, and at postoperative wk 1, 2, 4, 8, 16, and 24. Intravenous blood was drawn for WBC count and C-reactive protein quantification preoperatively, and during postoperative wk 1, 4, 16, and 24. MRI was performed on each knee at postoperative wk 8, 16, and 24. MRI protocols are outlined in the Supporting Information Text. At 24 wks, all animals were euthanized for further analysis. Vital organs (heart, lung, thyroid, liver kidneys, spleen, adrenal glands, testis) and relevant knee tissue (1 mm^3^ of repaired cartilage of left knee defects and adjacent tibial cartilage, synovium) were harvested and prepared for cell distribution RT-PCR. Chondral defects were prepared for further histological analysis and resultant histological results were further analyzed using O-Driscoll cartilage repair scores by 2 separate pathologists twice that did not participate in this study^11^. Synovial tissue of each knee harvested from anterior fat pads were also prepared for histological analysis and subsequent chronic synovitis grading in the same fashion^12^. For cell distribution analysis, PCR for human alu sequences was performed to confirm the presence of transplanted cartilage gels in recipient NHP organs. Total DNA of organ samples was extracted with a QIAamp DNA Mini Kit (Qiagen, Hilden, Germany), according to the manufacturer’s instructions. The primers used were Human Alu F: 5′-GTAAGAGTTCCGTAACAGAGCT-3′, Human Alu R: 5′-CCCCACCCTAGGAGAACTTCTCTTT-3′, NHP gapdh F: 5′-CGGATTTGGTCGTATTGGG-3′ and NHP gapdh R: 5′-GGGATCTCGCTCCTGGAAG-3′. Samples were incubated at 94 °C for 2 min and then amplified for 25 cycles of denaturation for 30 s at 94 °C, annealing for 30 s at 56 °C, and extension for 59 s at 72 °C. The PCR products were analyzed by resolution on a 1.5% (w/v)^57^. Cell tracking within the chondral defects were also performed using human anti-nuclei antibody MAB1281, clone 235-1 (Merck KGaA, Darmstadt, Germany) immunohistochemistry. Human anti-nuclei antibody (+) cells were counted and compared in 5 histological sections 20 μm apart for each defect. The transplantation site (cartilage defects) was also evaluated for CD45 expression, with IHC using anti-CD 45 antibody (Abcam, Cambridge, UK; catalog No. ab10558).

### Statistical analysis

Statistical analysis was performed using a software program (SPSS Version 18, Chicago, IL, USA). Non-parametrical tests were used throughout this study. The test is used as a non-parametric alternative of the independent two-sample t-test (Mann-Whitney) or multiple comparison (Kruskal-Wallis). Data are expressed as a mean ± standard deviation. P-values less than 0.05 were considered statistically significant^58^.

## Supplementary information

Supporting Information.

Supplemental online video 1.

Supplemental online video 2.
