# Supplementary material for: Engineered cartilage utilizing fetal cartilage-derived progenitor cells for cartilage repair
Source: Sci Rep. 2020 Mar 31;10:5722. doi: 10.1038/s41598-020-62580-0 (PMC7109068; doi:10.1038/s41598-020-62580-0)
Supplement: Supplementary file 1 — Supporting Information. [file 41598_2020_62580_MOESM1_ESM.doc]

Supporting Information

Title: Engineered cartilage utilizing fetal cartilage-derived progenitor cells for cartilage repair

Do Young Park, Byoung-Hyun Min*, So Ra Park, Hyun Ju Oh, Minh-Dung Truong, Mijin Kim, Ja-Young Choi, In-Su Park, Byung Hyune Choi

**Micro-CT analysis**

Volume of scaffold-engineered cartilage was analyzed and measured by a model 1076 X-ray micro-CT (SkyScan, Kontich, Belgium). Scanning was carried out with its resolution at pixel size of 18 µm (I = 190 µA, E = 40 kV, Filter = No filter). Scanned images were reconstructed and analyzed with commercial software (NRecon v.1.6.3.0, DataViewer v.1.4.4). At 1, 2 and 3 weeks, scaffold-engineered cartilages were compared with the detected volume by µCT.

**Polymerase chain reaction (PCR)**

RNA was extracted from cells using TRIzol (Invitrogen, Grand Island, NY) following the manufacturer’s instructions. Total RNA of 1 μg was reverse-transcribed into cDNA and 1 μg of cDNA was amplified using a first strand cDNA Synthesis Kit for RT-PCR (AMV, Roche, Mannheim, Germany) in the presence of specific primers. For real-time quantitative PCR, 1X SYBR Green Reaction Mix (Roche) was mixed with 20 ng cDNA and 10 pM of each primer. After initial incubation for 10 min at 95°C, the reactions were carried out for 30-40 cycles at 95°C for 10 s and 60°C for 30 s using a CFX96 Touch™ Real-time PCR Detection System (Bio-Rad, Hercules, CA). The relative gene expressions of the samples were normalized to GAPDH as an internal control and calculated by the comparative CT method. The primers used are shown in **Table S1**.

**Table S1.** Primers used in Real-Time PCR after the Reverse Transcription of mRNAs

| **Primers** | **Sequences** | **Length (bp)** | **Annealing Temp. (^o^C)** |
| --- | --- | --- | --- |
| GAPDH | F: 5’- GTATGTCGTGGAGTCTACTG -3’  R: 5’- GAGTTGTCATATTTCTCGTGGT -3’ | 150 | 60 |
| COL1A1 | F: 5’- GTCGAGGGCCAAGACGAAG -3’  R: 5’- CAGATCACGTCATCGCACAAC -3’ | 143 | 60 |
| COL2A1 | F: 5’- CGTCCAGATGACCTTCCTACG -3’  R: 5’- TGAGCAGGGCCTTCTTGAG -3’ | 122 | 60 |
| COL10A1 | F: 5’- ATATGGAGGTAGGCTGAA -3’  R: 5’- AGGTTTGTTGGTCTGATAG -3’ | 75 | 60 |
| ACAN | F: 5’- CTGCTTCCGAGGCATTTCAG -3’  R: 5’- CTTGGGTCACGATCCACTCC G-3’ | 98 | 60 |
| SOX9 | F: 5’-CTGAAGGGCTACGACTGGAC-3’  R: 5’-ACTGGTCTGCCAGCTTCCT-3’ | 139 | 60 |

**Table S2.** Cartilage repair score (from O’Driscoll et al. ^[1]^)

| Characteristics | Score |
| --- | --- |
| Nature of predominant tissue  Cellular morphology  Hyaline articular cartilage  Incompletely differentiated mesenchyme  Fibrous tissue or bone  Safranin-O staining of the matrix  Normal or nearly normal  Moderate  Slight  None  Structural characteristics  Surface regularity  Smooth and intact  Superficial horizontal lamination  Fissures 25–100% of the thickness  Severe disruption, including fibrillation  Structural integrity  Normal  Slight disruption, including cysts  Severe disintegration  Thickness  100% or normal adjacent cartilage  50–100% of normal cartilage  0–50% of normal cartilage  Bonding to the adjacent cartilage  Bonded at both ends of graft  Bonded at one end, or partially at both ends  Not bonded  Freedom from cellular changes of degeneration  Hypocellularity  Normal cellularity  Slight hypocellularity  Moderate hypocellularity  Severe hypocellularity  Chondrocyte clustering  No clusters  <25% of the cells  25–100% of the cells  Freedom from degenerative changes in adjacent cartilage  Normal cellularity, no clusters, normal staining  Normal cellularity, mild clusters, moderate staining  Mild or moderate hypocellularity, slight staining  Severe hypocellularity, poor or no staining | 4  2  0  3  2  1  0  3  2  1  0  2  1  0  2  1  0  2  1  0  3  2  1  0  2  1  0  3  2  1  0 |

**Nonhuman primate cartilage defect model**

A total of three chondral defects of 3mm diameter on the medial, lateral femoral condyles and trochlea were made in the right and left knee. The defects in the left knee received cartilage gel transplantation without additional fixation, while right knee defects served as controls. After wound closure, knees were immobilized with a cast for 1 week.

**MRI protocol for nonhuman primate cartilage defect model investigation**

The MRI images were acquired with a 3-T MR imaging unit (TrioTim; Siemens, Erlangen, Germany) with a six-channel cylindrical coil (Stark Contrasts, Erlangen, Germany) tailored for small animals, with an inner diameter of 7 cm. A sagittal 3D Multi-Echo Data Image Combination sequence (MEDIC) was obtained with the following parameters: repetition time msec/echo time msec, 54/22; flip angles, 12°; bandwidth, 233 Hz/pixel; field-of-view, 49 x 70 mm; section thickness, 1.0 mm; number of sections, 30; matrix, 256 x 180; number of signals acquired, two.; and total acquisition time, 8 minutes 3 seconds.

**Movie S1.** Video demonstrating the transplantation of the 2-wk cartilage gels into human chondral defects**:** The cartilage gels filled the defects and adhered to the defect without additional fixation and stayed within the defect even after considerable shaking in water.

**Movie S2.** Video demonstrating adhesiveness of the 2-wk cartilage gel: The adhesiveness of the cartilage gel is demonstrated in this video using a Compact Rheometer MCR102 (Anton Paar’s, Graz, Austria-Europe).


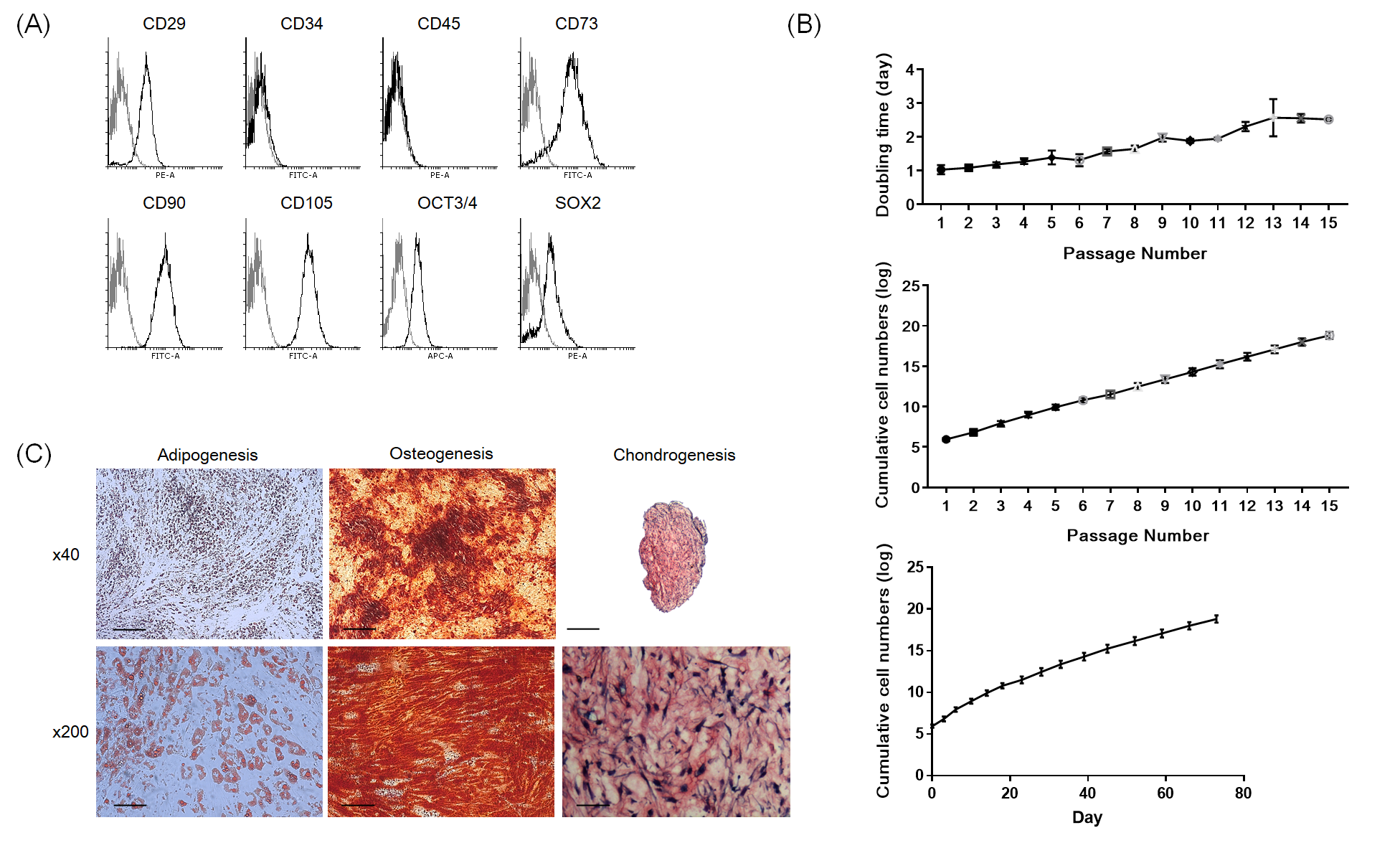


**Supplementary figure 1.** The basic characterization of fetal cartilage progenitor cells (FCPCs). (A) Expression of markers on FCPCs by flow cytometry. FCPCs at passage 2 had negative expression with CD34, CD45 and expression with CD29, CD73, CD90, CD105, OCT3/4 and SOX2. (B) We also examined the proliferation ability and phenotype of the FCPCs according to passage time. The doubling time of FCPCs was determined from passage 1 to passage 15 (n=4). Cells were subcultured at 80% confluence. The doubling time was calculted using the following formula: DT = (T1-T0)log2/(logN1-logN0), where T1-T0 = the culture period in days, N0 = the plating cell number, and N1 = the harvesting cell number. Accumulated cells numbers were calculated with passages or days. (C) For osteogenic and adipogenic differentiation, cells at passage 2 were evaluated by staining with Alizarin red S and Oil red O at 3 weeks of differentiation, respectively. For chondrogenic differentiation, the cell pellet was cultured in a chondrogenic medium and evaluted by staining with Safranin-O to observe the sulfated glycosaminoglycans after 3 weeks of induction. Magnification x40, x200.

Reference

[1] D. J. Moojen, D. B. Saris, K. G. Auw Yang, W. J. Dhert, A. J. Verbout*, Tissue E*n**g 20**02, 8, 627.
